# Prematurity-Related Hypertension in Children and Adolescents

**DOI:** 10.1155/2012/537936

**Published:** 2012-02-14

**Authors:** Karolina Poplawska, Karolina Dudek, Maja Koziarz, Dominik Cieniawski, Tomasz Drożdż, Sylwester Śmiałek, Dorota Drożdż, Przemko Kwinta

**Affiliations:** ^1^Department of Pediatrics, Polish-American Children's Hospital, Jagiellonian University Medical College, Wielicka 265 Street, 30-663 Cracow, Poland; ^2^Medical Student's Research Assosciation, Jagiellonian University Medical College, Wielicka 265 Street, 30-663 Cracow, Poland; ^3^Department of Pediatric Nephrology, Polish-American Children's Hospital, Jagiellonian University Medical College, Wielicka 265 Street, 30-663 Cracow, Poland

## Abstract

Due to the functional and structural immaturity of different organ systems, preterms have a higher rate of morbidity and mortality. The prevention and treatment of the complications of prematurity is a major challenge in perinatal health care. Recently, there have been several multicenter research trials analysing the impact of prematurity or low birth weight on the health problems of children and adolescents. Many of these studies deal with the issue of pediatric hypertension. An analysis of 15 studies conducted in the years 1998–2011, in which blood pressure values in ex-preterm children were measured, was performed. Comparison was based on several issues: measurement method, cohorts age, size, and birthweight. It has been proven that hypertension occurs more often in former preterm infants; however the etiologic pathways that cause this condition still remain unclear. Moreover, pediatric hypertension is a significant problem, because of its transformation into adult hypertension and increased cardiovascular risk later in life. Therefore it is crucial to introduce wide-spread screening and detection of elevated blood pressure, especially among prematurely born children.

## 1. Prematurity

Prematurity is defined as the birth of a child before 37 weeks of gestational age [1]. Infants born before 32 weeks are defined as “very preterm” and born before 28 weeks as “extremely preterm” newborns. It is estimated that the percentage of prematurely born children in most developed countries is about 5–7%. The prevalence in the United States is even higher, almost 13% [2]. 

There are many causes of preterm birth, such as spontaneous preterm labour, premature rupture of membranes, multiple gestations and assisted reproduction, eclampsia, intrauterine growth restriction, antepartum haemorrhage, cervical insufficiency, and uterine malformations. According to recent research studies, severe prematurity is mainly caused by infection or inflammation and abnormal vascular or placental development [3]. More than 50% of deliveries prior to 32 weeks have an inflammatory association [4].

Preterm birth is a major challenge in perinatal health care, mainly due to the various complications affecting these newborns. Advances in perinatal medicine have led to a continuous decrease in mortality of extremely low birth weight (ELBW) and extremely preterm infants [5]. Early complications (more severe in ELBW newborns) are respiratory distress syndrome, intraventricular hemorrhage, patent ductus arteriosus, and necrotizing enterocolitis and sepsis. The most dynamic complications occur in the first few weeks, but prematurity also influences health issues later in life. Late complications mainly include bronchopulmonary dysplasia retinopathy of prematurity [6–8]. Recently, there have been several multicenter research trials analysing the impact of prematurity or low birth weight on the health problems of children and adolescents. Many of these studies deal with the issue of hypertension (HT).

## 2. Hypertension in Ex-Preterm Newborns

A general description of studies from last 13 years in which blood pressure (BP) values in ex-preterm children were measured is presented in Table 1. In 8 out of 15 of the analyzed studies in addition to the measurement of BP values, the diagnosis of hypertension was also made [9–16].

Based on the age of the included subjects, studies can be divided into groups: evaluating school-aged children (6–12 years) [10–12, 16, 23], adolescents (13–18 years) [15, 17, 22], and adults (18–30 years) [9, 13, 14, 18–21]. The prevalence of hypertension in all studies ranged from 6 to 25%. In subjects from 6 to 12 years old the prevalence of hypertension was 10–25% [10–12, 16], in the adolescent group 16% [15], and in the adult group 6–10% [9, 13, 14]. According to this data it may seem like the prevalence of HT decreases with age. However it should be noted that these studies span the last 30 years during which advances in neonatal intensive care have increased the survival rates of ex-preterm infants exponentially. Before the 1970s only 10% of extremely low birth weight (ELBW) infants (500–999 g) or very preterm infants (<28 weeks gestational age) had a chance of surviving [24]. However in the late 1990s survival rates increased to almost 3-in-4 [25]. Therefore it is possible that the percentage of ELBW or very preterm survivors was lower in the adult group compared to the children's group. Could this mean that with increasing survival rates, the rate of long-term complications is rising as well? If this is true, since hypertension is a major risk factor of cardiovascular disease, which is one of the leading causes of morbidity and mortality in most countries, this could be a major public health problem in the following years. Information about the evolution of BP values in each group through the years would be most valuable, but there are no such trials concerning this issue at this time. In each study a different population was analyzed; therefore only the comparison of the presence of HT in different age groups was possible.

Taking into account the method of BP measurements, in 8 studies the authors used only casual BP measurements [9–11, 15, 17, 20, 21, 23]. In this group the prevalence of hypertension ranged from 10 to 24%. Ambulatory blood pressure monitoring (ABPM) alone was used in 2 studies [12, 16]. Hypertension in these cases was present in 10.3 [16] and 25% of children [12]. Both methods were used in 5 studies and hypertension frequency was between 6 and 9.3% [13, 14, 18, 19, 22]. When only one method of BP measurement was applied, the range of the percentage hypertension was larger than when both methods were used. It shows that HT is evaluated more precisely when both methods are used. Using ABPM reduces the risk of bias resulting from casual measurements. ABPM is a better diagnostic method but in some situations it cannot be appropriately performed in children which can also lead to misdiagnosing. Therefore these two methods seem to be complementary in assessing HT in children.

Another important aspect discussed in some studies is the influence of intrauterine growth restriction (IUGR) on the presence of HT in later life. No significant differences in BP values between small for gestational age (SGA) and appropriate for gestational age (AGA) preterms were detected in Keijzer-Veen et al. studies [9, 13]. In a Swedish study it was observed that full-term SGA children had significantly lower BP values than AGA preterms [18]. However in Shankaran et al. study the hypertension ratio was higher among full-term children with IUGR compared to controls [11]. In contradiction to this observation, a study from Israel showed that children with IUGR had lower BP values than their peers born without IUGR [23]. Because of the discrepancies between these results, it is difficult to clearly assess the impact of IUGR in preterm babies on BP values in future life.

Another approach to dividing studies is classification based on birthweight. Very low birth weight (VLBW) infants were included in 5 studies [14, 17, 19–21] and ELBW infants in 2 studies [10, 16]. In all of these studies results obtained in preterm children were compared to full-term infants. In studies on VLBW children the follow-up was conducted at the age of 15–27 years. Only one study assessed the presence of hypertension in VLBW children, and the prevalence was established as 9.3% [14]. Significantly higher systolic blood pressure (SBP) and/or diastolic blood pressure (DBP) in VLBW children was found in all of these studies [14, 17, 19–21]. Differences between subjects and controls were higher in SBP, ranged from 3.0 to 8.6 mmHg, but also substantial variations were observed in DBP, ranged from 3.5 to 5.3 mmHg between groups. With respect to studies with ELBW children follow-up was conducted at the school age (6–8 and 6–12 years). In both of the two studies the prevalence of hypertension was about 10%. There were no statistically significant differences between ELBW and full-term children as well as for SBP, DBP, and mean arterial pressure (MAP). Only one study revealed a significant difference between studied groups for systolic and diastolic BP load [10, 16]. Comparing the studies with VLBW and ELBW children, the main difference between them is the abundance of studied population (ranged from 44 to 195 versus 40 to 78). It may explain the lack of significance of results obtained in ELBW groups.

In 10 out of the 15 analyzed studies, SBP was significantly higher in former preterm newborns compared to controls regardless of the measurement method [12–20, 22]. Five studies revealed significantly elevated DBP in ex-preterms compared to controls [14, 16, 19, 21, 22]. MAP was significantly elevated in children in the 3 studies [18, 19, 22]. Furthermore, in all of the studies where ABPM was used, SBP was significantly elevated, but only two of these studies revealed significantly higher DBP [16, 22]. This data suggests that the main concern in preterm born children is systolic hypertension.

In diagnosing HT it is also important to differentiate the BP values during the day and night which is enabled by ABPM. In 4 of the analyzed studies, significant differences in daytime or nighttime SBPs values between ex-preterm children and controls were found. In one study former preterm children had significantly higher daytime SBP [13]. In another study only nighttime SBP was elevated [12]. Both daytime and nighttime SBPs were significantly elevated in 2 studies [19, 22]. For DBP there was no such correlation in any of the aforementioned studies.

Analysis of studies performed before 2008 revealed hypertension in 10–25% of ex-preterms, compared to 6–16% in studies from the last 3 years. Furthermore, in recent studies ABPM was used more frequently. There was no difference between patients' age and birth weight on follow-up. Based on the fact that through the years the diagnostic methods and criteria for HT in pediatric patients are more precise, children are less frequently overdiagnosed. This can explain the differences in HT prevalence between recent and older studies.

Six of the analyzed studies included patients from only one medical care unit. However, two of the one-center studies comprised of a large amount of study subjects (114 and 156 patients) [19, 22]. The prevalence of hypertension in these studies extended from 10 to 25% in former preterm children. More valuable studies are conducted nationwide due to their diversity and the limited influence of random variables on test results [9, 13]. In these studies the prevalence of hypertension was 6–10.3%. Studies involving large administrative areas of the country revealed a hypertension prevalence comparable to those nationwide and multicenter [14, 16, 17]. It is worth mentioning that in American multicenter studies the prevalence of hypertension was definitely higher (16–24%) than that in the corresponding European studies [11, 15]. The reason for this phenomenon might be the occurrence of more (besides prematurity) risk factors for HT development in American children such as black race and obesity.

To summarize, all the aforementioned studies were focused on different aspects of HT risk in ex-preterm children. The results are diversified and controversial in many cases. These discrepancies indicate the necessity of more multicenter and nationwide studies on larger populations. Conducting follow-ups in the same groups in intervals every few years during whole life might enable understanding of the problem of HT in prematurely born children.

## 3. Pathophysiology of Hypertension in Ex-Preterm Children

Impaired renal development in premature newborns has been proven to be related to hypertension later in life, however the etiologic pathways that cause this phenomenon still remain unclear. Impaired intrauterine kidney growth may lead to a reduction in the number of nephrons, which is characteristic for hypertensive patients' kidneys [26]. Recent studies have shown that in preterm infants glomerulogenesis as measured by radial glomerular counts was markedly decreased in comparison to term controls. Although the duration of glomerulogenesis is 40 days, the structure of preterm infants kidneys differs from that of the full-term infant [27]. Moreover, postnatal kidney growth of premature infants is impaired up until the second year of life [28, 29]. It is also worth mentioning that the use of nonsteroidal anti-inflammatory drugs during pregnancy and the newborn period can impair both renal structure and function [30]. A significant difference in renal volume has been proved to be present in ex-preterms at the age of 7, namely, relative right (86% ± 17 versus 99% ± 16 *P* < 0.001) and left (85% ± 19 versus 95% ± 18 *P* = 0.01) kidney volume in ex-preterms and controls, respectively [16].

Other theories assume that pathogenetic mechanisms such as increased tubular sodium channel expression, dysregulation of the intrarenal renin-angiotensin system, continuous oxidative stress, and decreased nitric oxide production leading to impaired endothelium-derived vasodilatation may play a role in inducing elevated BP in prematurely born infants [12, 31, 32].

Many studies emphasize the effect of metabolic imprinting on ex-preterm children and adolescents. The term “metabolic imprinting” refers to the basic biological phenomena that states that there is an underlying relationship between nutritional experiences early in life and chronic diseases later in life [33]. According to this theory, nutrition in early life has a major impact on health into early adulthood, notably on cardiovascular disease risk, bone health, and cognitive function. It has been shown in several studies that impaired nutrition in the perinatal period was linked to dysfunction of endothelium-dependent vasodilatation at birth and later in childhood [34, 35]. Several observations revealed that infants who were fed growth-promoting enriched diets had a greater risk of developing the main components of metabolic syndrome in the future. Greater nutrient intake in infancy is associated with raised BP and insulin resistance in adults [36, 37]. Multiple retrospective studies in diverse populations have shown that birth weight is inversely correlated with adult BP; thus the existence of a biological link between intrauterine growth and adult blood pressure is strongly suspected. Furthermore, several studies emphasize the effect of prenatal undernutrition on BP elevation. They have demonstrated that increased SBP was related to decreased maternal protein intake [33].

Another important point is the role of the sympathetic nervous system in the programming of hypertension [12]. In animal models, an increase in serum epinephrine levels inversely correlated to birth weight, which suggested an increase of adrenal medullary function [38]. It has also been shown that in SGA infants there is an increase in activity of the sympathetic component of the heart rate response. According to this data, it can be observed that the influence of the autonomous nervous system is programmed early in life. Being born SGA is associated with a greater risk of high blood pressure and other cardiovascular diseases in adulthood, especially where postnatal catch-up growth is apparent [39]. An American study analyzing the occurrence of prehypertension and hypertension in a group of former preterm infants at 16 years of age showed that an increased acceleration of weight gain between birth and 36 months was related to higher systolic and diastolic BP [15]. Since early weight gain catch-up has also been associated with obesity and insulin resistance in various studies [40, 41], it can increase the risk for cardiovascular disease later in life.

## 4. Target-Organ Damage

Recent data has shown that children and adolescents with a mild elevation in blood pressure are much more common than it was thought in the past [42]. Pediatric hypertension is a significant problem, because it frequently carries on into adult hypertension and increases the cardiovascular risk in later life [43–45]. Target organ abnormalities are commonly associated with hypertension in children and later in adults. This damage can be detected already even in young patients. In addition since there are no publications describing hypertension-related repercussions in preterm born children, we could suppose that they are exposed to similar complications of elevated BP as other children, for now, there are no documented discrepancies between the repercussions of hypertension in full- and preterm born children. In essential hypertension assessment of early organ damage provides a useful tool for treatment decisions [46].

The most frequently targeted organs damaged in childhood due to hypertension are the heart and blood vessels such as the coronary vessels, the cerebral vessels, and the vessels, in the kidney. Left ventricular hypertrophy (LVH) is the most remarkable sign of targeted organ damage caused by hypertension in children and adolescents [42]. The prevalence of LVH in children with hypertension varies from 14 to 42% [47–49]. The best tool to assess LVH is echocardiography, which should be performed after the diagnosis of HT has been made and periodically thereafter. The presence of LVH is one of the indications to initiate or intensify antihypertensive therapy [50]. Intima-media thickening (IMT) is another consequence of the effect of childhood hypertension on blood vessels. It is used to detect patients at a high risk for developing atherosclerosis in later life. Additionally arterial stiffness which is connected to IMT has been reported to be more frequent in hypertensive children [51]. Hypertension-related kidney damage is manifested by reduced renal function or Urinary Albumin Excretion (UAE). Microalbuminuria could be an early marker of kidney injury and also a predictor of LVH in children with essential hypertension [42, 52]. Impairment of cognitive function could be a result of narrowing of the small vessels in brain. It may also cause hypoperfusion and demyelination [53]. Retinal arteriolar narrowing is also a commonly known consequence of HT in children [46].

We could speculate that these changes occur earlier in ex-preterm children and adolescents, however there are no publications available on this subject at this time. It is essential to perform multicenter studies addressing the complications of hypertension in ex-preterm children.

## 5. Hypertension Risk Factors in Ex-Preterms

In unselected population of very young children (less than 6 years), hypertension is caused in most cases by renal parenchymal diseases such as glomerulonephritis, renal scarring, polycystic kidney disease, renal artery stenosis, and renal dysplasia [42]. These conditions and coarctation of the aorta together account for 70–90% of all cases of hypertension [46]. Over the age of 6 years more cases of essential hypertension start to occur.

It is a well-known fact that similar to adults, in children and adolescents, one of the most important risk factors of high BP is Body Mass Index (BMI), family history, and factors that the metabolic syndrome is composed of such as low plasma high-density lipoprotein, elevated plasma triglycerides, abdominal obesity, and insulin resistance [54–56]. Compared to their term sex- and age-matched lean counterparts, ex-preterms are affected by secondary hypertension much more frequently than essential. However, in numerous studies, significantly lower BMI values in ex-preterms have been confirmed, indicating that this group is thinner than controls, therefore diminishing the role of BMI, as a risk factor in this group of patients [21, 22]. Therefore in this group of patients, family history, BMI, and dyslipidemia are less important risk factors than those in the general pediatric population. It has been proven that obesity and being an ex-preterm infant are independent risk factors of hypertension [22].

## 6. Diagnostic Difficulties

Childhood hypertension is often asymptomatic or presents with uncharacteristic symptoms, such as headache, epistaxis, shortness of breath, and changes in behavior or school performance. Therefore it can be easily underdiagnosed. Diagnostic problems occur more often in children than they do in adults. These issues are common in all pediatric patients, regardless of their birth weight or prematurity. They are also habitually present in ex-preterms.

The first group of problems are technical issues related to proper BP measurement such as the appropriate BP cuff size and difficulty with auscultation in small children [53]. These problems occur more often in ex-preterms because they are often smaller than ex-terms and have a thinner arm. It is more difficult to match an appropriate BP cuff size to this group of patients. Secondly, in pediatric patients a necessary condition for proper BP measurement is the cooperation of the child. It has been proven that preterm infants are at a high risk of developing cerebral palsy. On the other hand, some ex-preterm infants are hyperactive which also make BP measurement difficult [57, 58]. Moreover, BP measurements can be overestimated more often in ex-preterms because of the children nervousness and hyperactivity.

The second group of problems, that both occur in ex-preterms and in ex-term infants alike, are two phenomena connected with hypertension: White Coat Hypertension (WCH) and masked hypertension. Only using office measurements of BP may be insufficient and could cause misdiagnosis and inappropriate treatment; thus a very useful method in the diagnosis of these phenomena is Ambulatory Blood Pressure Monitoring (ABPM) [59]. Ambulatory BP values are better markers of cardiovascular and renal risk than its office BP counterparts [60]. ABPM is also useful for evaluating patients with episodic hypertension, chronic kidney disease, diabetes, and autonomic dysfunction [50, 61]. Despite having many benefits, this method also has its limitations that are especially apparent in ex-pretem population. One of them is lack of reference values of AMBP in children with a height below 120 cm. An essential matter in children born with low birth weight (LBW) is that they are shorter and weigh less than children from the general population. Thus, a great number of these patients do not match ABPM criteria, which leads to the underdiagnosis of hypertension.

Therefore, further studies concerning children shorter than 120 cm should be conducted in order to generate AMBP standards in this group of patients. Though hard to execute, similar research studies in ex-preterm and IUGR infants would provide more accurate and earlier hypertension diagnosis.

## 7. Future Priorities

Regarding the increasing number of pediatric patients with hypertension and the severity of its complications, it is crucial to introduce wide-spread screening and detection of elevated blood pressure, especially among prematurely born children. Recent studies, comprising of prematurely born or LBW infants, clearly indicate a higher risk of hypertension in this group of patients [12, 13]. In consequence, future studies should focus on early and appropriate evaluation of the presence of high blood pressure values in former preterms. Furthermore, long-term, follow-up observations of BP should be performed in cohorts of ex-preterms aged 5, 10, 15, and 20 years old to determine BP elevation progression and its dynamics, the odds of developing hypertension, and the number of newly diagnosed hypertension cases in time. It could be a valuable extension of Lurbe et al. study concerning LBW newborns in which BP was measured during first year of life at 1, 3, 9, and 12 months of age [62]. It revealed that LBW infants had lower SBP and DBP at birth than normal weight children, but this relation was reversed after the 1st month. Because premature infants are at a greater risk for hypertension than full-term newborns, early restriction of its consequences is vital, and therefore further studies concerning this matter ought to be conducted. Due to lack of corresponding research studies in preterm infants, surveys regarding sodium intake reduction and ACE inhibitors implementation as possible alleviating factors of hypertension must be conducted [63, 64].

## Figures and Tables

**Table 1:** Selected clinical trials comparing BP values in different groups of children.

Study	Patients' age	Study population	Control group	Main conclusions
Pharoah et al. [17], United Kingdom, 1998	15 years	128 VLBW	128 NBW	The systolic blood pressure was significantly higher in cases than in controls. The diastolic pressure was also higher but the difference was not significant.
Kistner et al. [18], Sweden, 2000	26 years	33 (15 gestational week <32; 18 birth weight ≤2600 g)	17 NBW	Preterms had significantly higher casual systolic and mean arterial blood pressure levels compared to controls.
Doyle et al. [19], Australia, 2003	18 years	156 VLBW	38 NBW	BP was significantly higher in late adolescence in VLBW survivors than in NBW subjects. Growth restriction in utero was not significantly related to BP in VLBW survivors.
Hack et al. [20], United States, 2005	20 years	195 VLBW	208 NBW	VLBW individuals had a higher mean systolic blood pressure (SBP) than normal birth weight (NBW) control individuals (114 +/− 11 versus 112 +/− 13 mm Hg; *P* = 0.047). DBP did not differ between groups. The rates of hypertension did not differ significantly between the groups.
Keijzer-Veen et al. [9], Netherlands, 2005	19 years	588 (418 gestational week <32; 170 gestational week ≥32 and birth weight <1500 g)	—	The prevalence of hypertension is higher in individuals who were born preterm when compared with the general population. Birth weight SDS and gestational age both did not affect the risk for hypertension.
Rodriguez-Soriano et al. [10], Spain, 2005	6–12 years	40 ELBW	43 NBW	Systolic, diastolic, and mean blood pressures did not differ between cases and controls.
Shankaran et al. [11], United States, 2006	6 years	144 with IUGR	372 full-term	In term infants IUGR was linked to risk of hypertension in early childhood. Relative risk compared to control group was 1,8.
Bayrakci et al. [12], Turkey, 2007	5–17 years	41 preterms (<37 weeks of gestation)	27 full-term	There is an inverse correlation between high nighttime SBP SDS and birth weight. Lightness for date was discovered as a major predictor of both nocturnal and daytime SBP SDS. Nocturnal SBP SDS was closely correlated with 24 h HR SDS. 24 h HR SDS was elevated in nondippers. Preterms have increased nocturnal SBP resulting in greater frequency of nondippers.
Pyhälä et al. [21], Finland, 2009	23 years	44 VLBW	37 NBW	In comparison with the control group the VLBW group had higher systolic and diastolic blood pressure but the group differences were not statistically significant.
Lurbe et. al [22], spain, 2009	10–18 years	114 LBW	308 NBW	Obese low birth weight subjects had the highest systolic BP values over the 24 hours, whereas the nonobese subjects in the absence of low BW had the lowest values. No interaction existed between obesity and low birth weight in the office or ambulatory systolic blood pressure.
Keijzer-Veen et al. [13], Netherlands, 2010	20 years	50 very premature individuals <32 weeks of gestation (21 SGA, 29 AGA)	30 full-term	SGA had lower weight and height than AGA.there were No differences in BMI were found. In SGA more mothers had hypertension during pregnancy. Men had significantly higher SBP and lower HR. AGA–higher daytime SBP, baseline SBP, SBP load. SGA versus AGA. There were no differences in SBP, DBP, HF, and MAP loads. Newborns born very prematurely have higher SBPs, but IUGR has no effect on it.
Hovi et al. [14], Finland, 2010	18–27 years	118 VLBW	120 NBW	Higher rates of hypertension and higher 24-hour blood pressure in VLBW group were observed.
Vohr et al. [15], United States, 2010	16 years	296 (birth weight <1250 g)	95 NBW	The primary predictors of increased systolic blood pressure were weight gain velocity between birth and 36 months, pre-eclampsia, nonwhite race, and male gender. Predictors of diastolic blood pressure were weight gain velocity between birth and 36 months, brain injury, and male gender.
Fattal-Valevski et al. [23], Israel, 2011	8–12 years	64 IUGR (mean birth weight 1780 ± 422 g; 42% preterms)	64 NBW	Systolic blood pressure values were significantly lower in the IUGR group compared to the controls. There was no difference in diastolic blood pressure values. In the IUGR group, systolic blood pressure correlated significantly with current weight and body mass index, and diastolic blood pressure with weight gain between age 2 and 4 years. None of the blood pressure values correlated with birth weight.
Kwinta et al. [16], Poland 2011	6–7 years	78 ELBW	38 NBW	Hypertension was diagnosed in 10.3% of patient with ELBW. In control group hypertension was present in 5.2% cases but the difference was not significant. Statistically significant differences within night-time mean blood pressure, night-time blood pressure dipping, and mean systolic and diastolic BP load were detected

## Abbreviations

ABPM: Ambulatory blood pressure monitoring
AGA: Appropriate for gestational age
BMI: Body mass index
BP: Blood pressure
DBP: Diastolic blood pressure
ELBW: Extremely low birth weight
HT: Hypertension
IMT: Intima-media thickening
IUGR: Intrauterine growth restriction
LBW: Low birth weight
LVH: Left ventricular hypertrophy
NBW: Normal birth weight
MAP: Mean arterial pressure
SBP: Systolic blood pressure
SGA: Small for gestational age
UAE: Urinary albumin excretion
WCH: White coat hypertension
VLBW: Very low birth weight.
